# The First Evidence for the Role of *ACVR2A* Gene Fetal Genotype in Preeclampsia Susceptibility

**DOI:** 10.1002/mgg3.70069

**Published:** 2025-02-03

**Authors:** Asal Honarpour, Ahmad Majd, Hossein Sadeghi, Sayedhamid Jamaldini, Maryam Rahimi, Paniz Kazemzadeh, Reza Mirfakhraie

**Affiliations:** ^1^ Department of Genetics, Faculty of Biology Science North Tehran Branch, Islamic Azad University Tehran Iran; ^2^ Department of Biology, Faculty of Biology Science Islamic Azad University Tehran Iran; ^3^ Department of Medical Genetics, Faculty of Medicine Shahid Beheshti University of Medical Sciences Tehran Iran; ^4^ Medical Genomics Research Center, Tehran Medical Sciences Islamic Azad University Tehran Iran; ^5^ Department of Gynecology and Obstetrics, School of Medicine Iran University of Medical Sciences Tehran Iran; ^6^ Hematopoietic Stem Cell Research Center Shahid Beheshti University of Medical Sciences Tehran Iran

**Keywords:** *ACVR2*A, fetal genotype, polymorphism, preeclampsia

## Abstract

**Background:**

The *activin A receptor type 2A* gene (*ACVR2A*) plays an important role in normal gestation, particularly in decidualization, trophoblastic invasion, and placentation. Although several studies have investigated the association between *ACVR2A* maternal variants and preeclampsia (PE) susceptibility; however, controversial results were obtained. Moreover, in none of the previous studies, the role of *ACVR2A* fetal variants was explored. The aim of the present study was to investigate the role of *ACVR2A* rs1424954 and rs1424941 polymorphisms in PE susceptibility considering the impact of both fetal and maternal genotypes.

**Methods:**

For genotyping of *ACVR2A* rs1424954 and rs1424941, we performed TP‐ARMS‐PCR on 600 samples, including 400 peripheral blood samples from preeclamptic and normal women and 200 umbilical cord blood samples from each group of pregnant women.

**Results:**

Regarding rs1424954, only the fetal genotypes were associated with an increased risk of PE in both dominant and recessive inheritance models (OR = 2.88, 95% CI: 1.58–5.25, *p* = 0.0005; and OR = 2.43, 95% CI: 1.21–4.87, *p* = 0.012; respectively). For *ACVR2A* rs1424941variant, both maternal and fetal heterozygote genotypes were associated with PE susceptibility (OR = 1.57, 95% CI: 1.02–2.04, *p* = 0.04; and OR = 1.90, 95% CI: 1.02–3.54, *p* = 0.04; respectively).

**Conclusion:**

The present study confirmed the role of fetal *ACVR2A* polymorphisms in PE pathogenesis for the first time. However, replicated studies in diverse ethnicities are necessary to confirm the role of fetal genotype on susceptibility to PE.

## Graphical abstract

The effect of fetal genotype on preeclampsia (PE) is usually missed in the literature. We showed the first evidence for the role of fetal *ACVR2A* polymorphisms in PE.

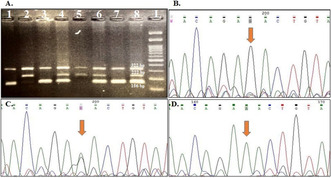

**Abbreviations**
ACVR2A: activin A receptor type 2A
BMI: body mass index
CIs: confidence intervals
HWE: Hardy–Weinberg equilibrium
IUGR: intrauterine growth restriction
ORs: odds ratios
PE: preeclampsia
SNP: single nucleotide polymorphism
TF: transcription factor
TP‐ARMS‐PCR: tetra‐primer amplification refractory mutation system–polymerase chain

## 1 Introduction

Preeclampsia (PE) is a severe common pregnancy disorder in humans. It could be generally defined as hypertension (systolic/diastolic blood pressure ≥ 140/90) and de novo proteinuria (> 300 mg in 24 h) after 20 weeks of gestation (Ankichetty et al. 2013; David‐Ona, De Castro, and Baltazar 2013; Do et al. 2018; Fitzpatrick et al. 2009; Gholami et al. 2018; Lambert et al. 2014). PE is a life‐threatening complication correlated with increased fetal and maternal mortality rates worldwide (Azimi‐Nezhad et al. 2020; Fitzpatrick et al. 2009; Sibai, Dekker, and Kupferminc 2005). It occurs in all main races and is developed in around 5% of pregnancies (Ferreira et al. 2015). PE is graded based on the severity to the following groups: severe PE (hypertension induced with proteinuria in addition to clinical signs such as headache, visual disturbances, and epigastric pain), mild PE (hypertension‐induced by pregnancy with proteinuria without any clinical signs which were found in the severe type), and eclampsia (severe PE with epileptic seizures). Clinical symptoms appear in the second trimester of gestation; however, primary pathogenic mechanisms appear very early (Ankichetty et al. 2013; Lambert et al. 2014). According to the various meta‐analyses and systematic review findings, the PE mechanism is supposed to be a combination of several factors such as environmental, biological, epigenetic, and genetic factors (Buurma et al. 2013; Ferreira et al. 2015; Giannakou, Evangelou, and Papatheodorou 2018). Genome extensive linkage analyses of PE pedigrees have shown several maternal susceptibility loci, located on chromosome 2, as strong positional candidate genes (Moses et al. 2006). According to the aim priority strategy, high priority is related to the *activin A receptor type 2*A gene (*ACVR2A*; OMIM: 102581) (Moses et al. 2006). *ACVR2A*, located at 2q22.3‐q23.1, consists of 12 exons and encodes activin A type 2A receptor that mediates the function of inhibin A, activin A, activin B, and myostatin. As a putative candidate gene, *ACVR2A* is a cell‐signaling protein known as a significant regulator of reproductive function (Fitzpatrick et al. 2009; Yanan et al. 2020). Activin A belongs to the TGF‐β (transforming growth factor) superfamily, a known biological indicator of PE. The elevated level of activin A in the maternal serum of preeclamptic women has been proven (Glotov et al. 2019). *ACVR2A* is important in establishing pregnancy via its effect on decidualization, trophoblastic invasion, and placentation (Thulluru et al. 2015). The first evidence regarding the association between *ACVR2A* polymorphisms and PE was reported by Moses et al. (2006), who showed a strong association for rs1424954. Since then, several replicated studies have been performed in different populations in which conflicting results were obtained (Ferreira et al. 2015; Glotov et al. 2019; Mendelova et al. 2018; Roten et al. 2009; Thulluru et al. 2015; Yanan et al. 2020; Zeybek et al. 2013). The medical evaluation of PE is mainly focused on maternal factors. However, fetal‐expressed genes may also play a role in trophoblast invasion and placentation. None of the previous studies considered the role of the fetal genotype in PE pathogenesis regarding *ACVR2A* gene. Therefore, we intended to evaluate the association of *ACVR2A* rs1424954 and rs1424941 variants with PE in Iranian patients by considering both the effects of fetal and maternal genotypes. To our knowledge, this is the first study investigating the role of fetal *ACVR2A* polymorphisms in PE pathogenesis.

## 2 Materials and Methods

### 2.1 Samples

A total of 400 pregnant women participated in this case–control study, including 200 healthy women (as controls) and 200 PE patients (as cases). Peripheral blood samples were drawn from each subject. Moreover, 200 umbilical cord blood samples, including 100 samples for each group of pregnant women, were collected immediately after birth to determine the fetal genotype. All the participants were recruited from the Akbar Abadi Hospital, Tehran, Iran. PE patients had resting systolic/diastolic blood pressure ≥ 140/90 and new onset of proteinuria after 20 weeks of gestation. They also had no history of personal hypertension before pregnancy. Healthy controls had no history of personal or familial PE. This research did not include individuals with a history of personal hypertension, renal disease, or proteinuria before pregnancy.

### 2.2 DNA Extraction and Genotyping

DNA was isolated from whole and cord blood samples using AnaCell Genomic DNA Extraction Kit (AnaCell, Iran) and stored at −20°C. We used a NanoDrop Spectrophotometer (Thermo Fisher Scientific) to assess the concentration and purity of the extracted DNA. Tetra‐primer ARMS PCR (TP‐ARMS‐PCR) was applied for genotyping *ACVR2A* gene rs1424954 and rs1424941 polymorphisms. Primers were designed using Primer1 online software available from http://primer1.soton.ac.uk/primer. The primers' specificity and characteristics were determined using the NCBI Primer‐Blast tool (available from https://www.ncbi.nlm.nih.gov/tools/primer‐blast/). The primer sequences for genotyping the *ACVR2* rs1424941 and rs1424954 polymorphisms are presented in Table 1.

**TABLE 1:** Primer sequences used for genotyping of *ACVR2* variants.

SNP	Primer	Primer sequence	Amplicon size (bp)
rs1424954	FI	ACCAAAACTTTGTAGAGTACATTAACATAG	156 (G allele)
	RI	TGACTGCCTTTCTCTTAAATACATTT	222 (A allele)
	FO	GCAGATAATTACAGGATGAACTTAATAGA	322
	RO	AAGGTAGAGATCTTGATTTTTTCCTTA	
rs1424941	FI	TGGTTGGTTTTTTAATTGTGCTAAAA	199 (A allele)
	RI	GTTACATTGTGAAGATCATAGTATGTCCC	295 (G allele)
	FO	TATGAATGTTTTAAAATCACAAAGCACA	439
	RO	GTAGAGGTTTCCTGAGGGAATAGATAAA	

For both rs1424954 and rs1424941 polymorphisms, each 20 μL PCR reaction mixture consisted of 1 μL of DNA sample (≥ 100 ng), 1.5 μL of each primer (10 Pmol), 12 μL of Taq DNA Polymerase 2X Master Mix RED (Amplicon, Korea), and 1 μL DNase‐free water, and PCR was performed in an Eppendorf thermocycler (Eppendorf, Germany). The cycling program consisted of 1 cycle of 95°C for 5′, 32 cycles including denaturation at 95°C for 30″, annealing at 55.7°C and 60°C for 50″ for rs1424954 and rs1424941, respectively, and extension at 72°C for 50″. For the final extension step, the PCR reactions were incubated at 72°C for an additional 10 min. The PCR products were separated on 2% agarose gel containing Gel Green Stain (Anna Cell, Iran) in 0.5X Tris/borate/EDTA (TBE) buffer. Moreover, 10% of samples were randomly selected and regenotyped by Sanger sequencing using an ABI 3730xl DNA analyzer. The obtained sequencing results were analyzed using Chromas software version 2.6.6.

### 2.3 In Silico Bioinformatics Analysis

We performed in silico functional analysis using RegulomeDB (http://www.regulomedb.org/), Ensembl genome browser (https://www.ensembl.org/), and HaploReg (https://pubs.broadinstitute.org/mammals/haploreg/haploreg.php) online tools to annotate the potential biological function of the studied variants.

### 2.4 Statistical Analysis

Deviation from Hardy–Weinberg equilibrium (HWE) was assessed by chi‐squared test using the SNPStats online software (https://www.snpstats.net/). The allele and genotype frequencies were also calculated using the same software. The association between *ACVR2* rs1424941 and rs1424954 genotypes and PE was determined using MedCalc version 20.100 online software (https://www.medcalc.org/). The strength of association between selected polymorphisms and PE susceptibility was calculated with Odds ratios (ORs) and 95% confidence intervals (CIs). A *p* < 0.05 was considered statistically significant.

## 3 Results

The mean age of the patient group was 29.94 ± 5.896, ranging from 17 to 42 years. In the control women, the mean age was 24.29 ± 5.873 with a range of 13–40 years. The demographic and clinical data of the PE patients and control individuals are presented in Table 2. As expected, systolic and diastolic blood pressure values were considerably higher in the PE patients compared to those measured in the control group (*p* < 0.0001). Body mass index (BMI) value was also significantly higher in PE patients than in healthy pregnant women (*p* < 0.0001). Moreover, fetal birth weight and gestational age at delivery were lower in the patients (*p* < 0.0001). Family history of hypertension was observed in 78 (39%) PE patients compared with 43 (21.5%) in healthy controls (OR = 2.33, 95% CI, 1.50–3.63, *p* = 0.0002). Among PE patients, 30 (15%) had a history of pregnancy loss compared to 14 (7%) in the control women (OR = 2.24, 95% CI, 0.89–5.61, *p* = 0.08).

**TABLE 2:** Comparison of the demographic data and clinical characteristics between the studied groups.

	Patients *N* = 200	Controls *N* = 200	*p*
Body mass index (kg/m^2^)	32.18 ± 5.171	28.21 ± 4.343	< 0.0001
M (IQR)	32.02 (28.57–35.00)	28.08 (25.06–30.43)	
Systolic blood pressure (mmHg)	152.2 ± 14.03	110.3 ± 7.769	< 0.0001
M (IQR)	150.0 (140.00–160.00)	110.0 (110.00–120.00)	
Diastolic blood pressure (mmHg)	96.50 ± 8.796	73.07 ± 7.286	< 0.0001
M (IQR)	90.00 (90.00–100.00)	70.00 (70.00–80.00)	
Gestational age (weeks)	36.01 ± 3.005	38.83 ± 1.018	< 0.0001
M (IQR)	37.00 (35.00–38.00)	39.00 (38.00–40.00)	
Fetal birth weight (kg)	2.842 ± 1.280	3.293 ± 0.4429	< 0.0001
M (IQR)	2.800 (2.21–3.26)	3.300 (3.00–3.60)	

*Note:* Values are shown as mean ± standard deviation.

Abbreviation: M (IQR): median (interquartile range).

Table 3 describes the distribution of maternal and fetal rs1424954 genotypes in the studied groups. Both maternal and fetal genotypes' frequencies were in HWE. No association was detected between maternal rs1424954 alleles and genotypes and PE risk regarding the maternal genotype; however, regarding the fetal genotype, the PE risk was increased in dominant and recessive modes of inheritance (OR = 2.88, 95% CI: 1.58–5.25, *p* = 0.0005; OR = 2.43, 95% CI: 1.21–4.87, *p* = 0.012; respectively). The rs1424954A allele was significantly higher in newborns of PE women and was associated with 2.24‐fold increased risk of PE.

**TABLE 3:** Genotype and allele distribution for maternal and fetal *ACVR2A* rs1424954 polymorphism.

Maternal rs1424954					
Model	Genotype/Allele	Case	Control	OR (95% CI)	*p*
	G/G	65 (32.5%)	76 (38%)	1	
	G/A	86 (43%)	85 (42.5%)	1.18 (0.76–1.85)	0.46
	A/A	49 (24.5%)	39 (19.5%)	1.47 (0.86–2.51)	0.16
Dominant	G/G	65 (32.5%)	76 (38%)	1	0.25
	A/G‐A/A	135 (67.5%)	124 (62%)	1.27 (0.84–1.92)	
Recessive	G/G‐A/G	151 (75.5%)	161 (80.5%)	1	0.23
	A/A	49 (24.5%)	39 (19.5%)	1.34 (0.83–2.16)	
Overdominant	G/G‐A/A	114 (57%)	115 (57.5%)	1	0.92
	A/G	86 (43%)	85 (42.5%)	1.02 (0.69–1.52)	
	G	216 (54%)	237 (59.25%)	1	0.13
	A	184 (46%)	163 (40.75%)	1.24 (0.94–1.64)	
**Fetal rs1424954**					
	G/G	25 (25%)	49 (49%)	1	1
	G/A	45 (45%)	36 (36%)	2.45 (1.28–4.70)	0.007
	A/A	30 (30%)	15 (15%)	3.92 (1.79–8.60)	0.0006
Recessive	G/A‐G/G	70 (70%)	85 (85%)	1	0.012
	A/A	30 (30%)	15 (15%)	2.43 (1.21–4.87)	
Dominant	G/G	25 (25%)	49 (49%)	1	0.0005
	G/A‐A/A	75 (75%)	51 (51%)	2.88 (1.58–5.25)	
Overdominant	G/G‐A/A	55 (55%)	64 (64%)	1	0.20
	G/A	45 (45%)	36 (36%)	1.45 (0.82–2.57)	
	G	95 (47.5%)	134 (67%)	1	0.0001
	A	105 (52.5%)	66 (33%)	2.24 (1.50–3.36)	

Abbreviations: CI, confidence interval; OR, odds ratio.

**FIGURE 1:**
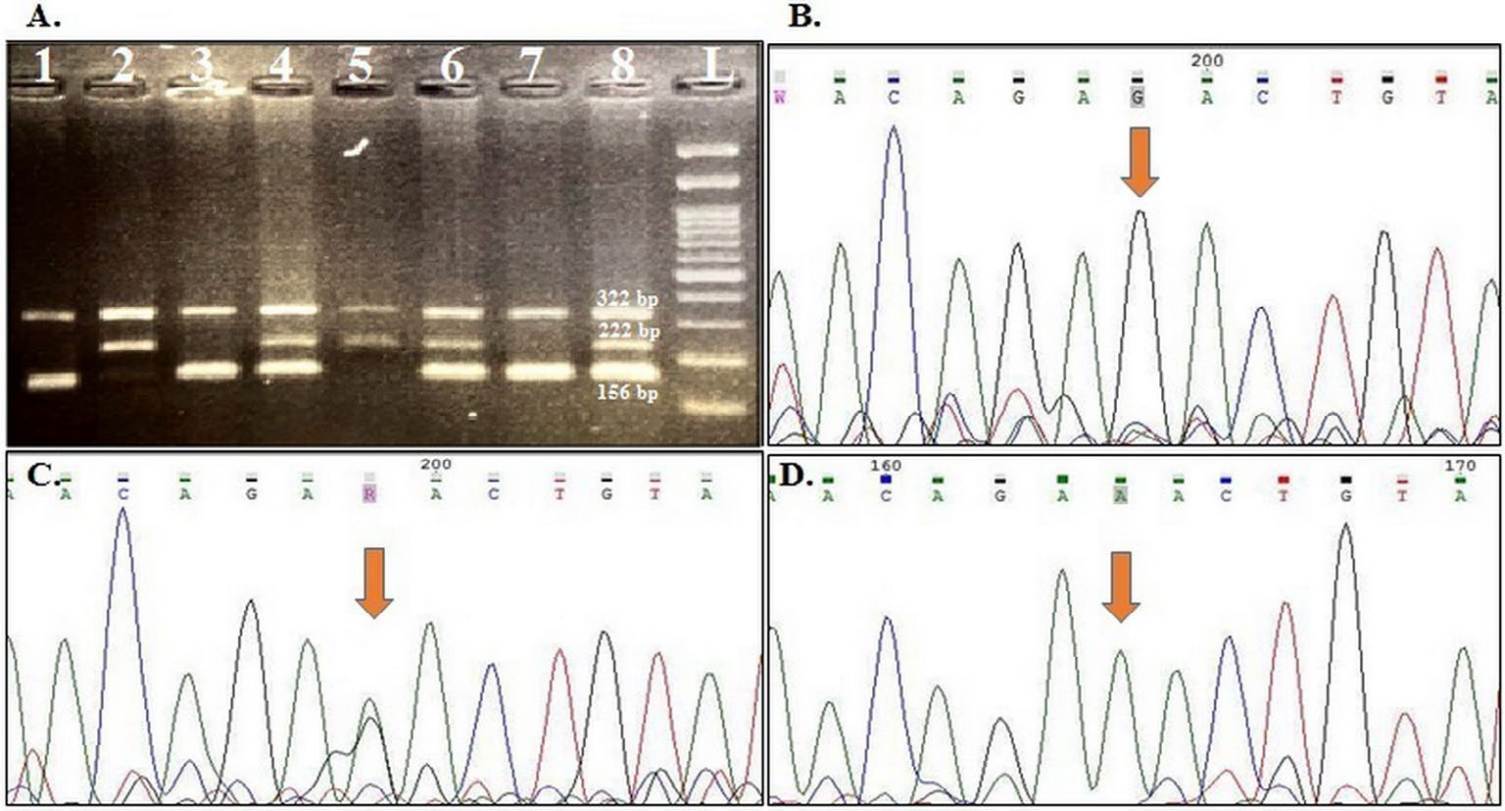
A sample of 2% agarose gel representing rs1424954 genotypes. Lanes 1, 3, and 7 show GG genotype; Lanes 4, 6, and 8 show GA genotype; Lanes 2 and 5 show AA genotype; and Lane L: 100 bp DNA ladder. Electropherograms show different rs1424954 genotypes, B: GG, C: GA, and D: AA.

**FIGURE 2:**
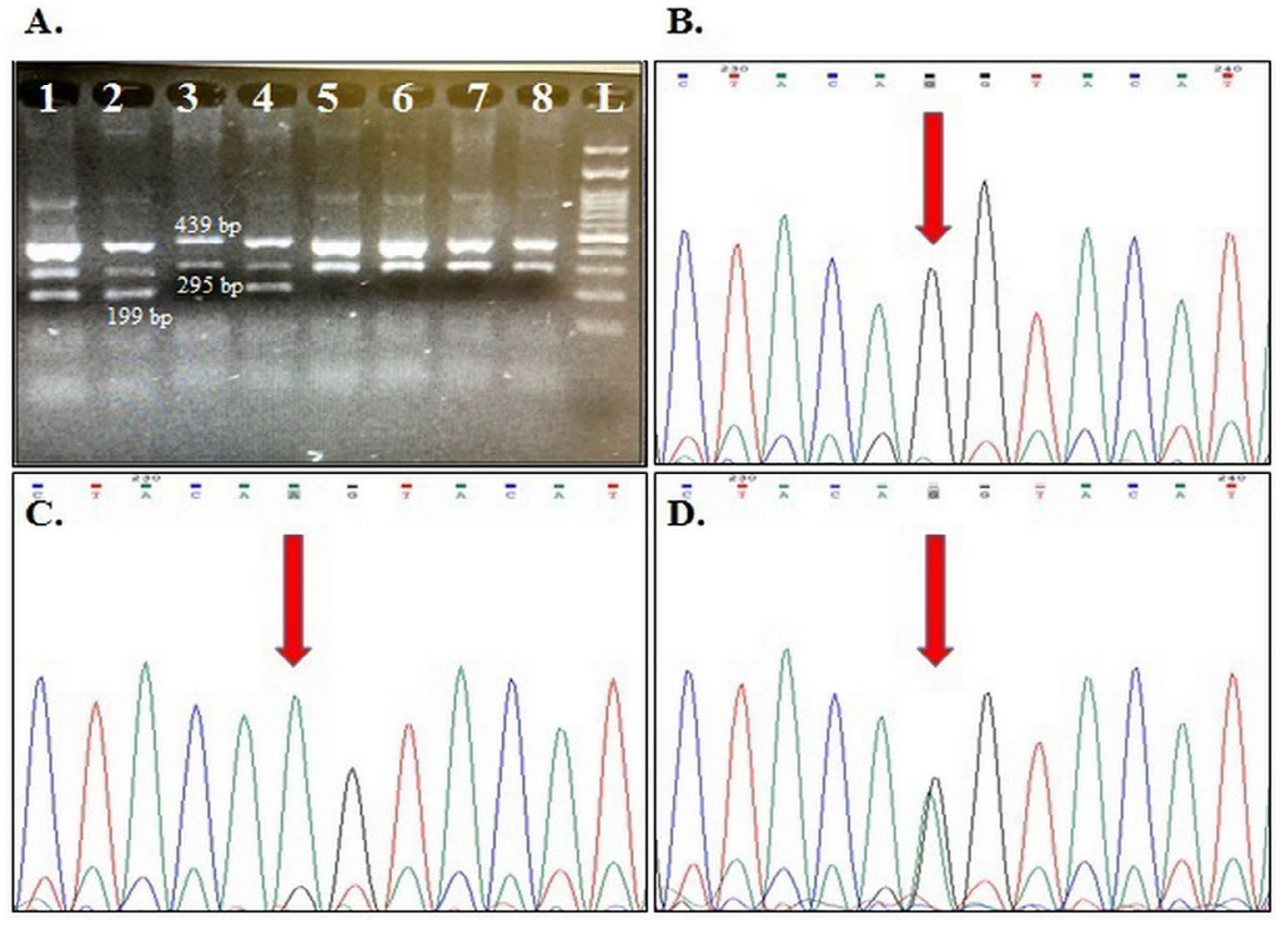
A sample of 2% agarose gel representing rs1424941 genotypes. Lanes 1, 2, and 4 show GA genotype; Lanes 3 and 5–8 show GG genotype; and Lane L: 100 bp DNA ladder. Electropherograms show different rs1424954 genotypes, B: GG, C: AA, and D: GA.

The genotype frequencies for rs1424941 are presented in Table 4. HWE was confirmed in all studied groups except for the distribution of maternal genotypes in healthy pregnant women. A weak association was detected between maternal and fetal heterozygote AG genotype and PE susceptibility in the studied samples (OR = 1.57, 95% CI: 1.02–2.04, *p* = 0.04; OR = 1.90, 95% CI: 1.02–3.54, *p* = 0.04; respectively). Although the frequency of maternal and fetal rs1424941A allele was higher in cases than in the related control groups, this difference was not statistically significant (*p* > 0.05). Figures 1 and 2 show gel electrophoresis patterns and the electropherograms representing different genotypes of *ACVR2A* rs1424954 and rs1424941 polymorphisms, respectively.

**TABLE 4:** Genotype and allele distribution for maternal and fetal *ACVR2A* rs1424941 polymorphism.

Maternal rs1424941					
Model	Genotype	Case	Control	OR (95% CI)	*p*
	G/G	117 (58.5%)	133 (66.5%)	1	
	G/A	73 (36.5%)	53 (26.5%)	1.57 (1.02–2.04)	0.04
	A/A	10 (5%)	14 (7%)	0.81 (0.35–1.90)	0.63
Dominant	G/G	117 (58.5%)	133 (66.5%)	1	0.099
	A/G‐A/A	83 (41.5%)	67 (33.5%)	1.41 (0.94–2.11)	
Recessive	G/G‐A/G	190 (95%)	186 (93%)	1	0.4
	A/A	10 (5%)	14 (7%)	0.70 (0.30–1.61)	
Overdominant	G/G‐A/A	127 (63.5%)	147 (73.5%)	1	0.031
	A/G	73 (36.5%)	53 (26.5%)	1.59 (1.04–2.44)	
	G	307 (76.75%)	319 (79.75%)	1	0.30
	A	93 (23.25%)	81 (20.25)	1.19 (0.85–1.67)	
**Fetal rs1424941**					
	G/G	61 (61%)	72 (72%)	1	1
	G/A	37 (37%)	23 (23%)	1.90 (1.02–3.54)	0.04
	A/A	2 (2%)	5 (5%)	0.47 (0.09–2.52)	0.38
Recessive	G/G	61 (61%)	72 (72%)	1	0.1
	G/A‐A/A	39 (39%)	28 (28%)	1.64 (0.91–2.98)	
Dominant	G/G‐G/A	98 (98%)	95 (95%)	1	0.26
	A/A	2 (2%)	5 (5%)	0.38 (0.07–2.08)	
Overdominant	G/G‐A/A	63 (63%)	77 (77%)	1	0.03
	G/A	37 (37%)	23 (23%)	1.97 (1.06–3.65)	
	G	159 (79.5%)	167 (83.5%)	1	0.30
	A	41 (20.5%)	33 (16.5%)	1.30 (0.79–2.17)	

Abbreviations: CI, confidence interval; OR, odds ratio.

In silico bioinformatics analysis revealed that *ACVR2A* rs1424954 and rs1424941 are located within transcription factor (TF) binding motifs. According to the HaploReg, rs1424954 is located at the binding motif for the POU3F2 TF, 1.3 kb upstream of the *ACVR2A* gene. RegulomeDB also assigned a rank of 4 for the rs1424954 variant, which confirms the TF binding evidence for this variant. The binding affinity for the A allele is approximately 12 times higher than for the G allele. Moreover, rs1424941 is an intronic variant and alters the affinity of the ZEB1 TF binding to its motif on the ACVR2A sequence by approximately 21‐fold.

## 4 Discussion

Previous studies regarding the association between *ACVR2A* polymorphisms and PE have only focused on the role of maternal genotype, and the role of fetal genotype is not well understood. The placenta is a fetomaternal organ, which means that fetal and maternal factors may affect a normal pregnancy and result in adverse pregnancy outcomes such as PE. In the current study, we investigated the association between *ACVR2A* polymorphisms, including rs1424954 and rs1424941, and PE risk in a sample of Iranian patients. Moreover, the effect of maternal and fetal genotypes on the disease susceptibility was considered. We found that both maternal and fetal rs1424941 heterozygote genotypes were associated with PE risk. Moreover, *ACVR2A* rs1424954 variant alleles and genotypes were significantly risk associated only in fetal samples. Several studies have investigated the role of maternal genotype *ACVR2A* polymorphisms in PE pathogenesis, although inconsistent results were reported. However, none of the previous studies investigated the role of fetal genotype on PE risk (Ferreira et al. 2015; Fitzpatrick et al. 2009; Lokki et al. 2011; Mendelova et al. 2018; Moses et al. 2006; Roten et al. 2009; Thulluru et al. 2015; Yanan et al. 2020; Zeybek et al. 2013).

Moses et al. were the first who define a PE susceptibility locus on chromosome 2 using positional cloning. They reported that *ACVR2A* gene rs1424954 was strongly associated with PE risk in the Australian/New Zealand population. Since then, the results obtained from the replicated studies in other populations have been inconsistent. While the association between PE and rs1424954 was confirmed in the Chinese population, other populations, including Norwegian, Northeastern Brazilian, and Finnish, failed to replicate the association (Ferreira et al. 2015; Lokki et al. 2011; Roten et al. 2009; Yanan et al. 2020). Moreover, although Ferreira et al. did not observe a statistically different distribution of rs1424954 genotypes between PE patients and controls, the variant was strongly associated with early‐onset PE (gestational age ≤ 34 weeks) (Ferreira et al. 2015). Deregulation of *ACVR2A* was previously reported in PE samples compared to the normal decidua tissues (Moses et al. 2006). Yong et al. also showed that the altered expression of decidual *ACVR2A* gene resulted in PE due to improper trophoblast function at the maternal–fetal interface and therefore abnormal placentation (Yong et al. 2018). SNPs, especially those located at the gene promoter region, may affect gene expression. Rs1424954 is located in the promoter region of *ACVR2A*, upstream of the transcription start site. Thulluru et al. investigated the effect of rs1424954 on *ACVR2A* expression to find probable relation between the susceptibility allele and PE. They showed that the existence of the G allele in the promoter region reduced the gene expression in trophoblastic cells compared to the A wild‐type allele. They concluded that *ACVR2A* expression reduction and pathological levels of activin A affect the trophoblast invasion (Thulluru et al. 2015). According to the HaploReg V4 (Ward and Kellis 2012), rs1424954 alters the motif affinity for binding the POU3F2 TF. POU3F2 is among TFs recently suggested to be downregulated in PE placental tissues (Zhang et al. 2020).

*ACVR2A* rs1424941 is an intronic variant, and the same as the present study, its association with PE was previously reported in the Norwegian population (Roten et al. 2009). However, the association was not observed in several populations, including Australian/New Zealand and Northeastern Brazilian (Ferreira et al. 2015; Fitzpatrick et al. 2009). We used the HaploReg v4.1 database to annotate the potential regulatory role of rs1424941 on *ACVR2A* expression and function (Ward and Kellis 2012). As an intronic variant, rs1424941 alleles alter the affinity of the ZEB1 TF for binding to its motif on the *ACVR2A* sequence. Interestingly, downregulation of ZEB1 TF was suggested to play an important role in trophoblast invasion and PE pathogenesis. Su et al. showed for the first time that aspirin mediated its preventive therapeutic effect on PE through TGF‐β1/ZEB1/miR‐200 signaling network (Su et al. 2021). Therefore, rs1424941 polymorphism, located at the ZEB1 binding motif, may be considered a pharmacogenetic target in future studies.

The other way that *ACVR2A* polymorphisms may contribute to PE pathogenesis is through binding to myostatin, the other member of the TGF‐β superfamily, as its receptor. It is proposed that myostatin plays an important role in the pathogenesis of several reproduction disorders, including PE, although the exact mechanism is not fully understood (Wang et al. 2022). Peiris et al. showed that the expression of myostatin was higher in women whose pregnancies were complicated by intrauterine growth restriction (IUGR) and PE (Peiris et al. 2015; Wang et al. 2022). It may be suggested that deregulation of *ACVR2A* due to its functional variants may affect the function of myostatin and contribute to PE pathogenesis; however, more studies are required to completely define the relationship among *ACVR2A*, myostatin, and PE development.

As mentioned earlier, we showed that fetal *ACVR2A* rs1424954 and rs1424941 variants were associated with PE risk, and *in silico* bioinformatics analysis also revealed that these variants may affect the expression of *ACVR2A* gene. Emerging evidence suggests the fetal genome's vital role in PE occurrence and development. An increased incidence of PE has been observed in pregnancies of Rubinstein–Taybi syndrome‐affected fetuses with EP300 mutations (van Voorden et al. 2023). Moreover, McGinnis et al. reported the first GWAS of offspring from PE pregnancies and showed that a variant in the fetal genome near FLT1 was associated with the disease risk (McGinnis et al. 2017). In a previous report, Amin‐Beidokhti et al. found that decreased expression of *Hsa‐miR‐517a/b* in the fetal side of placenta samples compared to maternal‐side tissue contributed to PE risk (Amin‐Beidokhti et al. 2021). Due to the important role of ACVR2A in establishing pregnancy via its effect on decidualization, trophoblastic invasion, and placentation, we may suggest that alteration in the gene expression on the fetal or maternal side of the placenta may contribute to the disease pathogenesis and progression.

The main limitations of the present study include (i) using much larger fetal and maternal samples may be worthwhile in evaluating such association with the disease subtypes and clinical variables; (ii) since the Iranian population is a mixed population consisting of several ethnic groups, sometimes population stratification along with a low sample size may cause deviation from HWE in the control group. Therefore, investigating the association between each ethnicity may be more informative. (iii) We have performed an *in silico* bioinformatics analysis to explain the possible role of the studied SNPs in PE development. The results of the *in silico* analysis need to be further confirmed by functional studies.

In conclusion, we showed for the first time that fetal *ACVR2A* variants contribute to the PE risk in our studied samples. Therefore, the present results may support the idea that fetal genotype is important in PE pathogenesis. More studies in different ethnicities with a larger sample size are required to confirm the role of fetal genes in PE development.

## Author Contributions

A.H., A.M., and R.M. contributed to the study conception and design. Samples collection, material preparation, and data analysis were performed by H.S., R.M., A.H., P.K., and M.R. The first draft of the manuscript was written by A.H. and S.H.J. and finally revised by R.M. All the authors read and approved the final manuscript.

## Ethics Statement

The present study was accomplished in accordance with the Declaration of Helsinki for research in human subjects (https://www.wma.net/policies‐post/wma‐declaration‐of‐helsinki‐ethical‐principles‐for‐medical‐research‐involving‐human‐subjects/). The study was also approved by the Shahid Beheshti University of Medical Sciences Ethics Committee (code: IR.SBMU.MSP.REC.1401.166). All subjects signed written informed consent prior to any experiment.

## Consent

The informed consent was taken from all the patients who participated in this study.

## Conflicts of Interest

The authors declare no conflicts of interest.

## Data Availability

The data that support the findings of this study are available from the corresponding author upon reasonable request.

## References

[mgg370069-bib-0001] Amin‐Beidokhti, M. , H. Sadeghi , R. Pirjani , L. Gachkar , M. Gholami , and R. Mirfakhraie . 2021. “Differential Expression of Hsa‐miR‐517a/b in Placental Tissue May Contribute to the Pathogenesis of Preeclampsia.” Journal of the Turkish German Gynecological Association 22, no. 4: 273–278. 34866368 10.4274/jtgga.galenos.2021.2021.0062 PMC8666996

[mgg370069-bib-0002] Ankichetty, S. P. , K. J. Chin , V. W. Chan , et al. 2013. “Regional Anesthesia in Patients With Pregnancy Induced Hypertension.” Journal of Anaesthesiology Clinical Pharmacology 29, no. 4: 435–444. 10.4103/0970-9185.119108. 24249977 PMC3819834

[mgg370069-bib-0003] Azimi‐Nezhad, M. , A. Teymoori , A. Salmaninejad , and R. Ebrahimzadeh‐Vesal . 2020. “Association of MTHFR C677T Polymorphism With Preeclampsia in North East of Iran (Khorasan Province).” Fetal and Pediatric Pathology 39, no. 5: 373–380. 10.1080/15513815.2019.1655819. 31448666

[mgg370069-bib-0004] Buurma, A. J. , R. J. Turner , J. H. Driessen , et al. 2013. “Genetic Variants in Pre‐Eclampsia: A Meta‐Analysis.” Human Reproduction Update 19, no. 3: 289–303. 10.1093/humupd/dms060. 23300202

[mgg370069-bib-0005] David‐Ona, D. , D. M. De Castro , and A. C. Baltazar . 2013. “The Distribution of Hypertension in the Philippine General Hospital After 4 Decades (A Comparative Study).” Acta Medica Philippina 47, no. 3: 49–52.

[mgg370069-bib-0006] Do, A. A. , E. Esmaeilzadeh , M. Amin‐Beidokhti , R. Pirjani , M. Gholami , and R. Mirfakhraie . 2018. “ACE Gene rs4343 Polymorphism Elevates the Risk of Preeclampsia in Pregnant Women.” Journal of Human Hypertension 32, no. 12: 825–830. 30127488 10.1038/s41371-018-0096-4

[mgg370069-bib-0007] Ferreira, L. C. , C. E. Gomes , A. C. Araújo , P. F. Bezerra , P. Duggal , and S. M. Jeronimo . 2015. “Association Between ACVR2A and Early‐Onset Preeclampsia: Replication Study in a Northeastern Brazilian Population.” Placenta 36, no. 2: 186–190. 10.1016/j.placenta.2014.11.007. 25499008

[mgg370069-bib-0008] Fitzpatrick, E. , M. P. Johnson , T. D. Dyer , et al. 2009. “Genetic Association of the Activin A Receptor Gene (ACVR2A) and Pre‐Eclampsia.” Molecular Human Reproduction 15, no. 3: 195–204. 10.1093/molehr/gap001. 19126782 PMC2647107

[mgg370069-bib-0009] Gholami, M. , R. Mirfakhraie , R. Pirjani , et al. 2018. “Association Study of FOXP3 Gene and the Risk of 0020 Pre‐Eclampsia.” Clinical and Experimental Hypertension 40, no. 7: 613–616. 29206055 10.1080/10641963.2017.1411500

[mgg370069-bib-0010] Giannakou, K. , E. Evangelou , and S. I. Papatheodorou . 2018. “Genetic and Non‐genetic Risk Factors for Pre‐Eclampsia: Umbrella Review of Systematic Reviews and Meta‐Analyses of Observational Studies.” Ultrasound in Obstetrics & Gynecology 51, no. 6: 720–730. 10.1002/uog.18959. 29143991

[mgg370069-bib-0011] Glotov, A. S. , S. V. Kazakov , E. S. Vashukova , et al. 2019. “Targeted Sequencing Analysis of ACVR2A Gene Identifies Novel Risk Variants Associated With Preeclampsia.” Journal of Maternal‐Fetal and Neonatal Medicine 32, no. 17: 2790–2796. 10.1080/14767058.2018.1449204. 29506428

[mgg370069-bib-0012] Lambert, G. , J. F. Brichant , G. Hartstein , V. Bonhomme , and P. Y. Dewandre . 2014. “Preeclampsia: An Update.” Acta Anaesthesiologica Belgica 65, no. 4: 137–149. 25622379

[mgg370069-bib-0013] Lokki, A. I. , M. M. Klemetti , S. Heino , L. Hiltunen , S. Heinonen , and H. Laivuori . 2011. “Association of the rs1424954 Polymorphism of the ACVR2A Gene With the Risk of Pre‐Eclampsia Is Not Replicated in a Finnish Study Population.” BMC Research Notes 4: 545. 10.1186/1756-0500-4-545. 22177086 PMC3267796

[mgg370069-bib-0014] McGinnis, R. , V. Steinthorsdottir , N. O. Williams , et al. 2017. “Variants in the Fetal Genome Near FLT1 Are Associated With Risk of Preeclampsia.” Nature Genetics 49, no. 8: 1255–1260. 28628106 10.1038/ng.3895

[mgg370069-bib-0015] Mendelova, A. , V. Holubekova , M. Grendar , et al. 2018. “Association Between 3'UTR Polymorphisms in Genes ACVR2A, AGTR1 and RGS2 and Preeclampsia.” General Physiology and Biophysics 37, no. 2: 185–192. 10.4149/gpb_2017028. 29593124

[mgg370069-bib-0016] Moses, E. K. , E. Fitzpatrick , K. A. Freed , et al. 2006. “Objective Prioritization of Positional Candidate Genes at a Quantitative Trait Locus for Pre‐Eclampsia on 2q22.” Molecular Human Reproduction 12, no. 8: 505–512. 10.1093/molehr/gal056. 16809377

[mgg370069-bib-0017] Peiris, H. N. , H. Georgiou , M. Lappas , et al. 2015. “Expression of Myostatin in Intrauterine Growth Restriction and Preeclampsia Complicated Pregnancies and Alterations to Cytokine Production by First‐Trimester Placental Explants Following Myostatin Treatment.” Reproductive Sciences 22, no. 10: 1202–1211. 10.1177/1933719115572482. 25736326

[mgg370069-bib-0018] Roten, L. T. , M. P. Johnson , S. Forsmo , et al. 2009. “Association Between the Candidate Susceptibility Gene ACVR2A on Chromosome 2q22 and Pre‐Eclampsia in a Large Norwegian Population‐Based Study (The HUNT Study).” European Journal of Human Genetics 17, no. 2: 250–257. 10.1038/ejhg.2008.158. 18781190 PMC2696227

[mgg370069-bib-0019] Sibai, B. , G. Dekker , and M. Kupferminc . 2005. “Pre‐eclampsia.” Lancet 365, no. 9461: 785–799. 10.1016/s0140-6736(05)17987-2. 15733721

[mgg370069-bib-0020] Su, M. T. , P. Y. Tsai , C. Y. Wang , H. L. Tsai , and P. L. Kuo . 2021. “Aspirin Facilitates Trophoblast Invasion and Epithelial‐Mesenchymal Transition by Regulating the miR‐200‐ZEB1 Axis in Preeclampsia.” Biomedicine & Pharmacotherapy 139: 111591. 10.1016/j.biopha.2021.111591. 33865015

[mgg370069-bib-0021] Thulluru, H. K. , O. J. Michel , C. B. Oudejans , and M. van Dijk . 2015. “ACVR2A Promoter Polymorphism rs1424954 in the Activin‐A Signaling Pathway in Trophoblasts.” Placenta 36, no. 4: 345–349. 10.1016/j.placenta.2015.01.010. 25659497

[mgg370069-bib-0022] van Voorden, A. J. , R. Keijser , G. J. Veenboer , et al. 2023. “EP300 Facilitates Human Trophoblast Stem Cell Differentiation.” Proceedings of the National Academy of Sciences of the United States of America 120, no. 28: e2217405120. 37406095 10.1073/pnas.2217405120 PMC10334808

[mgg370069-bib-0023] Wang, S. , L. Fang , L. Cong , J. P. W. Chung , T. C. Li , and D. Y. L. Chan . 2022. “Myostatin: A Multifunctional Role in Human Female Reproduction and Fertility—A Short Review.” Reproductive Biology and Endocrinology 20, no. 1: 96. 10.1186/s12958-022-00969-4. 35780124 PMC9250276

[mgg370069-bib-0024] Ward, L. D. , and M. Kellis . 2012. “HaploReg: A Resource for Exploring Chromatin States, Conservation, and Regulatory Motif Alterations Within Sets of Genetically Linked Variants.” Nucleic Acids Research 40(Database issue): D930–D934. 10.1093/nar/gkr917. 22064851 PMC3245002

[mgg370069-bib-0025] Yanan, F. , L. Rui , L. Xiaoying , et al. 2020. “Association Between ACVR2A Gene Polymorphisms and Risk of Hypertensive Disorders of Pregnancy in the Northern Chinese Population.” Placenta 90: 1–8. 10.1016/j.placenta.2019.11.004. 31790936

[mgg370069-bib-0026] Yong, H. E. J. , P. Murthi , B. Kalionis , R. J. Keogh , and S. P. Brennecke . 2018. “Decidual ACVR2A Regulates Extravillous Trophoblast Functions of Adhesion, Proliferation, Migration and Invasion In Vitro.” Pregnancy Hypertension 12: 189–193. 10.1016/j.preghy.2017.11.002. 29203340

[mgg370069-bib-0027] Zeybek, B. , H. A. Celik , H. H. Aydin , and N. Askar . 2013. “Polymorphisms in the Activin A Receptor Type 2A Gene Affect the Onset Time and Severity of Preeclampsia in the Turkish Population.” Journal of Perinatal Medicine 41, no. 4: 389–399. 10.1515/jpm-2012-0187. 23633461

[mgg370069-bib-0028] Zhang, H. , Y. He , J. X. Wang , et al. 2020. “miR‐30‐5p‐Mediated Ferroptosis of Trophoblasts Is Implicated in the Pathogenesis of Preeclampsia.” Redox Biology 29: 101402. 10.1016/j.redox.2019.101402. 31926626 PMC6928320

